# Medical student wellness in the United States during the COVID-19 pandemic: a nationwide survey

**DOI:** 10.1186/s12909-021-02837-y

**Published:** 2021-07-26

**Authors:** Louis Nikolis, Andrea Wakim, William Adams, Prempreet Bajaj DO

**Affiliations:** 1 Loyola University Chicago Stritch School of Medicine, 2160 South First Avenue, 60153 Maywood, Illinois USA; 2 Departments of Medical Education and Public Health Sciences, Chicago Health Sciences Division, Loyola University, 2160 South First Avenue, 60153 Maywood, Illinois USA; 3 Department of Orthopaedic Surgery and Rehabilitation, Loyola University Medical Center, 2160 South First Avenue, 60153 Maywood, Illinois USA

**Keywords:** COVID-19, medical education, medical students, public health crises, wellness

## Abstract

**Background:**

As United States (US) medical students suffer higher rates of depression and anxiety than the general population, the wellness of medical students is particularly salient. One definition describes wellness as having eight dimensions: Intellectual, emotional, physical, social, occupational, financial, environmental, and spiritual. As the coronavirus (COVID-19) pandemic poses unique challenges for medical students, we aimed to compare medical student wellness before and during the COVID-19 pandemic.

**Methods:**

An informal survey was created to assess eight wellness dimensions and was distributed via Survey Monkey to US allopathic and osteopathic medical students via email and social media. The survey was administered from March 29, 2020 to June 23, 2020. Univariable and multivariable linear mixed-effects models were used to estimate the change in students’ overall wellness using an 11-point scale ranging from 0 (least well) to 10 (most well). Generalized estimating equations were used to estimate the change in students’ responses to the eight dimensions before and during the COVID-19 pandemic.

**Results:**

On multivariable analysis, students reported a decline in their overall wellness during COVID-19 (*M*_diff_ = -1.08; *p* < .001). Asian respondents reported little change in overall wellness (*M* = -0.65) when compared to White respondents (*M* = -1.16) and Black respondents (*M* = -1.57). Students felt less supported and comfortable with their social (*OR* = 0.47) and daily (*OR* = 0.45) environments and expressed decreased satisfaction with their exercise (*OR* = 0.85), sense of purpose (*OR* = 0.33), and financial status (*OR* = 0.75). Students also expressed lower confidence (*OR* = 0.15) and satisfaction (*OR* = 0.11) with their medical education and increased anxiety (*OR* = 3.37) and depression (*OR* = 2.05).

**Conclusions:**

Medical students reported declines in overall wellness and individual wellness dimensions. These findings can be used to implement changes to improve medical student wellness.

**Supplementary Information:**

The online version contains supplementary material available at 10.1186/s12909-021-02837-y.

## Background

Compared to the general population, United States (US) medical students have significantly higher rates of depression, anxiety, burnout, and suicidal ideation [1–3]. Medical students suffering from depression and burnout are more prone to academic and professionalism struggles, decreased empathy, suicidal ideation, and withdrawing from medical school than their peers [3–8]. Factors which contribute to these higher rates of distress in medical students include personality, physical activity levels, coping strategies, social support, student loans, and life stressors both in the preclinical/clinical settings and outside of medicine [4, 8–12]. Given these alarming statistics, the relationship between medical education and student wellness is increasingly important to both students and medical school professionals. Many US medical schools offer well-being activities and curricula for students, yet few schools directly define and assess student wellness [8]. One definition describes wellness as having eight dimensions: Intellectual, emotional, physical, social, occupational, financial, environmental, and spiritual [13, 14]. This model of wellness has been used to guide departmental wellness programs during the coronavirus (COVID-19) pandemic and has been adapted by the Substance Abuse and Mental Health Services Administration for their wellness initiative [14, 15]. These eight dimensions intertwine to define one’s overall wellness [13, 14].

The COVID-19 pandemic has posed new and unique challenges for medical students. As a result of national and institutional mandates such as stay-at-home orders, students are grappling with curricular and lifestyle changes that affect their future careers and current daily lives. On top of their taxing education, these challenges place students at higher risk for reduced wellness.

Few studies have examined the pandemic’s effects on medical student wellness. One study found that psychological wellbeing and work performance were decreased in medical students in Saudi Arabia during the pandemic [16]. Another study found that medical students in India had worse mental health during COVID-19, including higher anxiety and stress levels, which were linked to poor sleep quality [17]. Though these studies examined some components of wellness, no published studies have examined the eight dimensions of wellness, particularly in US medical students. Analyzing medical student wellness during this unique time may highlight ways to improve the structure of medical education and promote wellness in the future. Therefore, the aim of our study was to administer a single survey to measure US medical student wellness before and during the COVID-19 pandemic.

## Methods

The authors used eight dimensions of wellness as a guideline to create an informal, *de novo* survey (see Supplementary File 1, .pdf, Medical Student Wellness During the COVID-19 Pandemic, which is a copy of the survey). The survey was sent via Survey Monkey to medical education coordinators, student affairs leaders, and medical student wellness groups from allopathic and osteopathic schools across the US, who were asked to share it with medical students at their institutions. Additionally, the survey was shared on social media sites, including Facebook, Twitter, and Instagram by posting on medical school class pages, medical student specific accounts, and using medical student related hashtags (i.e. #MedStudent and #MedStudentTwitter). Responses were collected from March 29, 2020 through June 23, 2020, and participation was completely voluntary. Informed consent was obtained from participants. Respondents were asked to complete the survey only one time, and incomplete survey responses were included in the analysis. The study was submitted to the Institutional Review Board (IRB) for the Protection of Human Subjects at Loyola University Chicago Health Sciences Division before beginning any study procedures and received IRB exemption.

### Survey Design

Our survey collected basic demographic information (i.e., year in school, sex, race, name of medical school), information regarding current education status (participation in recorded lectures vs. live-online lectures), and subjective information regarding each of the eight wellness domains. Students were asked to respond to the survey items labeled “during COVID-19” based on their current state of wellness and to respond to items labeled “before COVID-19” by recalling their feelings before the pandemic.

The survey contained 28 items pertaining to wellness. The creation of these items was guided by the definitions of eight wellness domains with six items covering physical wellness, and four items covering each of the intellectual and emotional wellness domains; three items each were used to assess social, occupational, and spiritual wellness, while two items assessed financial and environmental wellness. A single item assessed overall wellness. Seventeen of the 28 items were items that capitalized on an ordinal (Likert) scale ranging from ‘strongly disagree’ to ‘strongly agree.’ Six items were questions regarding frequency of activities (e.g., How many days do you exercise per week?). Two items were ‘select all that apply’ questions (e.g., What strengthens your sense of purpose?). Two items were multiple choice questions (e.g., Where do you spend the majority of your time during the week?). One item asked students to rate their overall wellness before and during COVID-19 on a scale from 0 to 10. The final survey question was an optional free response where students could share any comments that were not addressed in the survey.

### Statistical Methods

Respondent characteristics were reported as valid counts and proportions stratified by the COVID incidence rate of the state they were living in at the time of the survey based on “Trends in Cumulative Incidence Rate of COVID-19 Cases Reported to CDC.” [18] The date when the cumulative incidence data was collected coincided with the last day of survey administration, which was June 23, 2020. The respondent was considered living in a high incidence state if he/she lived in a state with a single day incidence rate per 100,000 of 800 or greater, while moderate incidence rate was between 500–800, and low incidence rate was considered 500 or lower.

Regarding overall wellness, univariable and multivariable linear mixed-effects models were used to estimate the average decline in overall wellness from their pre-pandemic response. The multivariable model adjusted this decline for respondents’ sex, year in medical school, race, their state’s incidence rate, whether they were assisting with COVID-19 relief efforts at the time of survey as well as their medical school characteristics including whether they were taking a required and/or elective class or clerkship, whether their classes or clerkships used recorded and/or live online lectures, and whether their classes or clerkships required examinations. In these models, we specified a completely general (unstructured) covariance matrix and allowed random intercepts for each school and for each respondent within each school to account for the hierarchical design (within-subject correlation). For the multivariable model, multicollinearity diagnostics were monitored for tolerance, variance inflation, and shared variance proportions. Residual plots showed no violations of linearity or homoscedasticity.

Students also answered questions about their medical school training, sleep and nutrition/exercise behaviors, social activities, finances, and mood using ordinal (Likert-type) scales. For these items, generalized estimating equations (GEE models) were used to compare the odds of a higher response on the scale during versus pre-pandemic. Each model specified a multinomial distribution with cumulative logit link for the ordered response categories, and an exchangeable working correlation matrix was used to account for students’ paired (correlated) responses. While robust standard errors were used to construct 95 % confidence limits for the effect sizes (odds ratios), these comparisons were not subjected to null hypothesis tests. A similar approach was used to compare the odds of respondents spending most of their time at home rather than elsewhere during the pandemic, though this model specified a binomial distribution with logit link for the response. Finally, all remaining survey questions were *check-all-that-apply* items. For each question, we used exact McNemar chi-square tests to compare the proportion checked during versus pre-COVID. All analyses were completed using SAS version 9.4 (Cary, NC).

## Results

Because As medical education administrators, coordinators, and other educators were asked to distribute the survey to their medical students (i.e., the survey employed snowball sampling), the exact response rate is not estimable. However, there were a total of 1,389 respondents from 38 states. Among these individuals, 11 (0.8 %) were excluded because they did not list their academic institution, and one (0.07 %) was excluded because he was living outside the United States at the time of the survey.

The remaining 1,377 respondents were from 112 medical schools in the United States. While approximately 17 % (n = 231) of the responses came from the authors’ institution, the remaining 83 % (n = 1, 146) were well dispersed among the remaining 111 medical schools in the sample. Most lived in a state with a high (n = 629 or 46 %) COVID-19 incidence rate at the time of survey; only 20 % (n = 272) were living in a state with a moderate COVID-19 incidence rate, while another 35 % (n = 476) were living in a state with a low COVID-19 incidence rate. Most identified as White (n = 908 or 66 %), female (n = 922 or 67 %), and were in their first or second year of medical school (n = 729 or 53 %); another 33 % (n = 459) of respondents were in their third year of medical school, while few (n = 189 or 14 %) reported that they were in their fourth year of medical school. Nearly every respondent reported that their school suspended in-person meetings (n = 1,375 or 99 %), and the majority reported that they were taking a required class or clerkship (n = 1,049/1,376 or 76 %) that capitalized on recorded (n = 1,004/1,363 or 74 %) as well as live online lectures (n = 1,058/1,365 or 78 %). As expected, the majority reported that their class or clerkship required examinations (n = 1,129/1,365 or 83 %). Finally, at the time of the survey, few participants reported they were taking an elective class or clerkship (n = 407/1,371 or 30 %) or were actively involved in COVID-19 relief efforts (n = 508/1,376 or 37 %). In fact, a sparse number of respondents were providing in-patient care (n = 50/1,376 or 3.6 %) or caring for COVID-19 patients (n = 27/1,376 or 2.0 %). See Table 1.

**Table. 1:** Demographic information of medical student respondents

	COVID Incidence Rate			Total (*N* = 1377)
	**Low(*n* = 476)**	**Moderate(*n*** **=272)**	**High(*n* = 629)**	
**Year in medical school**				
MS1	131 (28 %)	79 (29 %)	165 (26 %)	375 (27 %)
MS2	135 (28 %)	69 (25 %)	150 (24 %)	354 (26 %)
MS3	141 (30 %)	91 (33 %)	227 (36 %)	459 (33 %)
MS4	69 (15 %)	33 (12 %)	87 (14 %)	189 (14 %)
**Sex**				
Female	319 (67 %)	187 (69 %)	416 (66 %)	922 (67 %)
Male	156 (33 %)	85 (31 %)	208 (33 %)	449 (33 %)
Non-binary	1 (0.2 %)	0	4 (0.6 %)	5 (0.4 %)
Prefer not to say	0	0	1 (0.2 %)	1 (0.1 %)
**Race or ethnicity**				
White	335 (70 %)	188 (69 %)	385 (61 %)	908 (66 %)
Hispanic or Latino	33 (6.9 %)	11 (4.0 %)	36 (5.7 %)	80 (5.8 %)
Black or African American	10 (2.1 %)	25 (9.2 %)	24 (3.8 %)	59 (4.3 %)
Asian	72 (15 %)	34 (13 %)	134 (21 %)	240 (17 %)
Native American	1 (0.2 %)	0	1 (0.2 %)	2 (0.2 %)
Other	21 (4.4 %)	9 (3.3 %)	37 (5.9 %)	67 (4.9 %)
Prefer not to say	4 (0.8 %)	5 (1.8 %)	12 (1.9 %)	21 (1.5 %)
**School suspended in-person meetings**	474 (99 %)	272 (100 %)	629 (100 %)	1375 (99 %)
**Taking required class or clerkship** (*N = 1376*)	376 (79 %)	186 (69 %)	487 (77 %)	1049 (76 %)
**Taking elective class or clerkship** (*N = 1371*)	128 (27 %)	83 (31 %)	196 (31 %)	407 (30 %)
**Classes or clerkships utilize recorded lectures** (*N = 1363*)	369 (78 %)	189 (72 %)	446 (72 %)	1004 (74 %)
**Classes or clerkships utilize live online lectures** (*N = 1365*)	392 (83 %)	227 (86 %)	439 (70 %)	1058 (78 %)
**Classes or clerkships have required exams** (*N = 1365*)	396 (84 %)	198 (75 %)	535 (86 %)	1129 (83 %)
**Currently assisting with in-person patient care** (*N = 1376*)	16 (3.4 %)	18 (6.6 %)	16 (2.5 %)	50 (3.6 %)
**Currently providing in-person care of COVID-19 patients** (*N = 1376*)	10 (2.1 %)	5 (1.9 %)	12 (1.9 %)	27 (2.0 %)
**Currently assisting with COVID-19 relief efforts?** (*N = 1376*)	179 (38 %)	103 (38 %)	226 (36 %)	508 (37 %)

*Note*: Unless otherwise noted, the number of respondents = 1377 from 112 medical schools.

Using a numeric response scale (range 0:10) where higher scores indicate better overall wellness, students’ average pre-pandemic wellness score was 6.95 (SD = 1.53), and their average pandemic wellness score was 5.87 (SD = 2.01). This represented an approximate decline of one point in overall wellness (*M*_diff_ = -1.08, 95 % CI: -1.21 to -0.95; *p* < .001) even after controlling for students’ sex, year in medical school, race, whether they were assisting with COVID-19 relief efforts at the time of survey, their state’s COVID incidence rate at the time of survey, and select medical school characteristics including whether they were taking a required and/or elective class or clerkship, whether their classes or clerkships used recorded and/or live online lectures, and whether their classes or clerkships required examinations. See Table 2.

**Table. 2:** Change in overall wellness of medical student respondents

	Unadjusted		Adjusted	
	**Mean Difference(95 % CI)**	***p***	**Mean Difference(95 % CI)**	***p***
**Pandemic vs. Pre-Pandemic Wellness**	-1.077 (-1.203 to -0.952)	< 0.001	-1.080 (-1.208 to -0.952)	< 0.001

*Note*: The number of respondents used to compute the unadjusted estimate = 1377. The number of respondents used to compute the adjusted estimate = 1,326. The multivariable estimate is adjusted for respondents’ binary sex, year in medical school, race (i.e., recoded as White, Asian, Black, Hispanic, or Other), their state’s ordinal incidence rate of COVID at the time of survey (i.e., Low, Moderate, or High), whether students were assisting with COVID-19 relief efforts at the time of survey. and their binary medical school characteristics including whether they were taking a required and/or elective class or clerkship; whether their classes or clerkships used recorded and/or live online lectures, and whether their classes or clerkships required examinations

Sensitivity analyses revealed this change score *nominally* depended on respondents’ race (interaction *p* = .01), whether they were taking an elective class or clerkship (interaction *p* = .001), and whether their schools used live online lectures (interaction *p* = .03). That is, students identifying as Asian reported little change in overall wellness (*M*_diff_ = -0.65, 95 % CI: -0.95 to -0.35; *p* < .001) when compared to White respondents (*M*_diff_ = -1.16, 95 % CI: -1.32 to -1.01; *p* < .001) and Black respondents (*M*_diff_ = -1.57, 95 % CI: -2.18 to -0.96; *p* < .001). Similarly, students taking an elective class or clerkship reported little change in overall wellness (*M*_diff_ = -0.73, 95 % CI: -0.97 to -0.50; *p* < .001) when compared to those *not* taking an elective class or clerkship (*M*_diff_ = -1.22, 95 % CI: -1.37 to -1.07; *p* < .001); and students using live online lectures reported little change in overall wellness (*M*_diff_ = -1.00, 95 % CI: -1.15 to -0.86; *p* < .001) when compared to those *not* using live online lectures (*M*_diff_ = -1.35, 95 % CI: -1.63 to -1.08; *p* < .001). In this sample, no other respondent or school characteristic was associated with overall wellness.

For the Likert scale items, students reported sleeping more during the pandemic (*OR* = 3.25, 95 % CI: 2.80–3.77) and that their sleep was more satisfactory (*OR* = 1.26, 95 % CI: 1.12–1.43). Despite their increased sleep and quality of sleep, however, students also reported lower levels of energy during the pandemic (*OR* = 0.28, 95 % CI: 0.24–0.32). Regarding students’ nutrition and exercise behaviors, they reported exercising more frequently during COVID-19 (*OR* = 1.25, 95 % CI: 1.11–1.41) but also reported lower satisfaction with their exercise regimen (*OR* = 0.85, 95 % CI: 0.74–0.98) and lower overall satisfaction with their nutritional intake (*OR* = 0.73, 95 % CI: 0.65–0.83). Further, students reported less satisfaction with their social environment during COVID-19. For example, even though students spent more time (days) engaging with their family each week (*OR* = 2.10, 95 % CI: 1.89–2.34), they also reported low levels of support from their social environment (*OR* = 0.47, 95 % CI: 0.42–0.53) and low overall comfort with their daily environment (*OR* = 0.45, 95 % CI: 0.39–0.52). Similarly, students reported low satisfaction with their sense of purpose (*OR* = 0.33, 95 % CI: 0.29–0.36) despite spending substantially more time reflecting on their sense of purpose (*OR* = 3.28, 95 % CI: 2.96–3.63). Also, compared to their pre-COVID response, students spent more time (days) worrying about their finances (*OR* = 1.71, 95 % CI: 1.59–1.85) and expressed less satisfaction with their finances (*OR* = 0.75, 95 % CI: 0.70–0.79). See Table 3 (also see Supplementary File 2, .docx, Summary frequencies for Table 3, which indicates summary frequencies for ordinal scale questions with baseline values pre-COVID and differences during COVID).

**Table. 3:** Odds of a higher survey response during vs. pre-COVID from medical student respondents

	Valid N	Odds Ratio	95 % CI	
			**Lower**	**Upper**
**Physical**				
Hours sleeping per night	1321	3.25	2.80	3.77
Satisfaction with sleep	1288	1.26	1.12	1.43
High energy levels	1288	0.28	0.24	0.32
Satisfaction with nutritional intake	1288	0.73	0.65	0.83
Days exercising per week	1321	1.25	1.11	1.41
Satisfaction with exercise	1288	0.85	0.74	0.98
**Social**				
Days speaking with family per week	1321	2.10	1.89	2.34
Supported by social environment	1288	0.47	0.42	0.53
**Spiritual**				
Days reflecting on sense of purpose	1321	3.28	2.96	3.63
Satisfaction with sense of purpose	1288	0.33	0.29	0.36
**Environmental**				
Comfort with daily environment	1288	0.45	0.39	0.52
**Financial**				
Days worrying about finances	1321	1.71	1.59	1.85
Satisfaction with financial status	1288	0.75	0.70	0.79
**Intellectual**				
More hours studying per day	1321	0.99	0.90	1.10
Enough time to study	1288	3.96	3.43	4.56
Worry about grades	1288	1.12	1.03	1.22
Confidence in medical education	1288	0.15	0.13	0.17
**Occupational**				
Comfort in providing patient care	1286	0.30	0.27	0.33
Satisfaction with work/school	1288	0.11	0.09	0.13
Enjoyment of work/schoolwork	1288	0.25	0.22	0.28
**Emotional**				
Higher stress	1288	2.98	2.59	3.42
Higher anxiousness	1288	3.37	2.99	3.81
Higher depression	1288	2.05	1.88	2.23
Higher burn out	1288	1.60	1.43	1.80

Table 3 also shows that students spend more time studying each week during COVID-19 (*OR* = 3.96, 95 % CI: 3.43–4.56) but are increasingly concerned about their grades (*OR* = 1.12, 95 % CI: 1.03–1.22) and lack confidence in their medical education (*OR* = 0.15, 95 % CI: 0.13–0.17), satisfaction with their training (*OR* = 0.11, 95 % CI: 0.09–0.13), and enjoyment of their schoolwork (*OR* = 0.25, 95 % CI: 0.22–0.28). Students also expressed higher levels of stress (*OR* = 2.89, 95 % CI: 2.59–3.42), anxiety (*OR* = 3.37, 95 % CI: 2.99–3.81), depression (*OR* = 2.05, 95 % CI: 1.88–2.23), and burn out (*OR* = 1.60, 95 % CI: 1.43–1.80) compared to their pre-COVID response.

Additionally, students were less likely to endorse schoolwork (58 % vs. 84 %), friends or family (80 % vs. 82 %), hobbies (51 % vs. 55 %), or community service (32 % vs. 51 %) as activities that strengthen their sense of purpose during COVID. Instead, they were more likely to endorse reflection (45 % vs. 38 %) or other activities (4.1 % vs. 2.1 %) as strengthening their sense of purpose. See Table 4.

**Table. 4:** Summary of responses to tertiary survey questions by medical student respondents

	Before COVID	During COVID
**Spend majority of time**		
At a friend or significant other’s home	10 (0.8 %)	58 (4.4 %)
At home	194 (15 %)	1221 (92 %)
At school or work	1083 (82 %)	26 (2.0 %)
Other	15 (1.1 %)	4 (0.3 %)
Outdoors	19 (1.4 %)	12 (0.9 %)
Total	1321 (100 %)	1320 (100 %)
**Method of interaction with friends/family**		
In person^a^	1119 (81 %)	544 (40 %)
Text^a^	1235 (90 %)	1193 (87 %)
Facetime application^a^	645 (47 %)	1104 (80 %)
Phone-call^a^	1048 (76 %)	1074 (78 %)
Email^a^	248 (18 %)	274 (20 %)
**Strengthens sense of purpose**		
School/work^a^	1155 (84 %)	797 (58 %)
Friends/family^a^	1133 (82 %)	1097 (80 %)
Hobbies^a^	762 (55 %)	696 (51 %)
Community service^a^	709 (51 %)	443 (32 %)
Religion^a^	398 (29 %)	379 (28 %)
Reflection^a^	518 (38 %)	615 (45 %)
Other^a^	36 (2.1 %)	56 (4.1 %)

*Note*: ^a^Item requested a check-all-that-apply response. These items treat missing responses as unchecked. Therefore, the valid *N* = 1377 for each item

Regarding the optional free response survey item, there were 185 respondents. Approximately 21 % (n = 39) of these respondents commented on stress regarding United States Medical Licensing Examination (USMLE) Step 1 and Step 2 and Comprehensive Osteopathic Medical Licensing Examination of the United States (COMLEX-USA) Level 1 and Level 2. One student wrote, “Everything being so up in the air has greatly increased my anxiety, in particular Step 1. I have had a very hard time getting myself to study. I have family in a very high-risk area and I am constantly concerned for them, and this has made me behind in my studies to the point that now I have a lot of anxiety regarding that as well.“ Another student wrote, “The uncertainty around Step scheduling and how the school is handling communications is contributing greatly to my anxiety and decreased sense of purpose and motivation.” Additionally, 9.7 % (n = 18) of respondents commented on disappointment with their school’s administration. For example, one student wrote “Lack of empathy, timely communication, and solutions by school admin and national institutions (usmle, prometric) is extremely disheartening.“ Another respondent stated, “Our school is not responsive to feedback. They do not want to make an effort to change. They are more concerned with the appearance of caring for our wellness rather than actual productive concern that would result in changes.”

## Discussion

The unique and unprecedented circumstances of the pandemic are potential driving forces for the observed changes in medical student wellness. Among all respondents, there was a significant decrease in overall wellness during the COVID-19 period. Further, sensitivity analyses did reveal significant differences in overall wellness depending on respondents’ race. Of note, when compared to Asian respondents, Black and White respondents reported a more significant decline in overall wellness. Studies have shown that Black students are particularly susceptible to the effects of discrimination as race is strongly linked to self-identity, which may explain why Black respondents experience greater reductions in wellness during COVID comparatively[19]. Interestingly, other studies have shown that burnout is more common among students in the majority (rather than minority), similar to our findings of White respondents reporting significant declines in overall wellness as a consequence of COVID [20]. Therefore, additional research is required to further investigate the relationship between race and wellness in medical students.

During the time of survey administration, nearly all medical students were removed from in-person classroom and clinical settings, per AAMC Guidelines [21]. As a result, many schools transitioned to virtual learning in an attempt to replace traditional medical training. However, studies have shown that there are many barriers to virtual medical education, including inadequate implementation of technical skills, insufficient resources, and lack of institutional guidance and peer support [22, 23]. Despite these known difficulties with virtual learning and an inherently different caliber of medical education during the pandemic, most medical schools did not provide adjustments to tuition, which costs an average of $50,201 annually [24]. As approximately 73 % of medical students have educational debt and graduate medical school with $200,000 of debt, the challenges of virtual learning may exacerbate students’ financial concerns [25]. Therefore, the transition to online learning may contribute to the significant reductions in intellectual, occupational, and financial wellness found in this study, including less confidence in medical education, less comfort in providing patient care, less satisfaction with work/schoolwork, less financial satisfaction, and worrying about finances more frequently.

Further, removing students from clinical experiences could hinder academic and personal growth. In previous emergency crises, including Hurricane Rita, the AIDS Epidemic, and the attack on September 11, 2001, US medical students assisted on the frontlines and learned valuable lessons regarding critical decision-making and emergency care [26, 27]. However during the initial months of the pandemic, students could not participate in similar and irreplaceable learning opportunities, which may have been disappointing and contrary to their personal missions in medicine [28]. Likewise, students on their clinical rotations could not interact with or “carry their own” patients, a valuable responsibility and learning opportunity [29]. Similarly for pre-clinical students, in-person experiences such as volunteering in hospitals, shadowing physicians, and partaking in Objective Structured Clinical Examinations to practice clinical skills were prohibited or were virtual. This could explain the noted reductions in intellectual and occupational wellness in first through fourth year medical students.

Further, a medical student’s personal identity is strongly tied to their professional identity in medicine and service, studies have shown that identity roles contribute to one’s sense of purpose [29–31]. This professional identity formation begins to develop as early as the first year of medical school [32]. Therefore, restricting students from assisting on the frontlines, removing students from the clinical environment and in-person experiences, and labeling students as “non-essential” offer explanations for their reduced spiritual wellness, including decreased satisfaction with their sense of purpose.

Other dramatic changes that occurred during the COVID-19 period include changes regarding the scheduling of national licensing exams, such as USMLE Step 1 and 2 and COMLEX-USA Level 1 and 2. Students across the country had these exams rescheduled, canceled, or postponed [33]. As highlighted by many respondents of the free response survey item, these exams are already a significant source of stress and anxiety, and these changes could have led to reductions in emotional wellness, including increased stress, anxiety, depression, and burnout [34, 35]..

The residency application process also changed due to COVID-19, as visiting student rotations were cancelled (unless meeting an AAMC Exception) and residency interviews occurred virtually as opposed to in-person [36]. This step is crucial for career planning and is a source of anxiety. Some students may excel during in-person interviews where they can interact with the program director, faculty, current residents, and other candidates. However, with a virtual platform, these interactions may be less organic or not be possible. This new platform could also explain increased stress and anxiety. Moreover, for fourth year students, cancellation of celebratory events like Match Day and graduation may have played a role in their worsened emotional wellness.

Societal changes induced by COVID-19 have affected medical students as well. Although studies suggest that reduced contact hours and increased free time to explore one’s interest could in fact increase levels of wellness, with quarantine and social distancing orders in place, students spent more time at home with less in-person interactions [37]. Stay-at-home orders and closures of local businesses may limit one’s physical activity and foster a sedentary lifestyle [38]. Sedentary lifestyles are associated with numerous adverse health outcomes, including obesity, depression, and anxiety [39–41]. This may help explain the reduced physical and emotional wellness found in this study, including lower energy, less satisfaction with nutrition and exercise, and increased feelings of depression and anxiety.

To minimize COVID-19 exposures, on-campus meetings and courses were limited or nonexistent, removing medical students from their classmates, usual study groups, and social environment. Studies have shown that study groups, social interaction, and student communities are important for medical students from both social and educational perspectives [42, 43]. This may explain the observed reductions in social and environmental wellness, including students feeling less supported by their social environment and less comfortable in their daily environments.

### Limitations

Our study is not without limitations. While we aimed to minimize sampling bias by contacting administrators from all US allopathic and osteopathic schools, the survey distribution was largely dependent on each individual medical school’s survey policy. Additionally, distribution via social media was limited to those who use social media and saw the postings and was thus another component of sampling bias. For these reasons, the response rate was not estimable, and the number of respondents from each school varied. Another limitation of our study included non-response bias. It is possible that those who completed the survey have different opinions than those who did not. Additionally, COVID-19 restrictions varied across states and across time, which may have impacted respondents differently during our survey distribution period. Students may have responded differently based on their perceptions of their pre-pandemic wellbeing, as students were asked during the pandemic to indicate their wellness pre-COVID and during COVID. Furthermore, our informal survey was not validated or piloted prior to its use. Still, the survey responses provided by medical students throughout the country provide insight into their resiliency, frustrations, and overall experiences as a consequence of the COVID-19 pandemic.

## Conclusions

This is the first study examining wellness amongst US medical students during the COVID-19 pandemic. Our study demonstrates that during the COVID-19 time period, medical students reported lower levels of wellness overall and in all eight dimensions: Intellectual, emotional, physical, social, occupational, financial, environmental, and spiritual. Our findings shed light on the vulnerability of medical student wellness. Further research is needed to evaluate targeted strategies to improve and promote medical student wellness during times of crises.

## Supplementary Information

**Additional file 1:**

**Additional file 2:**

## Data Availability

The datasets used and/or analyzed during the current study are available from the corresponding author on reasonable request.

## References

[1] Goebert D, Thompson D, Takeshita J (2009). Depressive symptoms in medical students and residents: a multischool study. Acad Med J Assoc Am Med Coll.

[2] Lloyd C, Gartrell NK (1984). Psychiatric symptoms in medical students. Compr Psychiatry.

[3] Dyrbye LN, Thomas MR, Massie FS (2008). Burnout and suicidal ideation among U.S. medical students. Ann Intern Med.

[4] Dyrbye L, Shanafelt T. A narrative review on burnout experienced by medical students and residents. Med Educ. 2016;50(1):132–49. 10.1111/medu.12927. 10.1111/medu.12927 26695473

[5] Thomas MR, Dyrbye LN, Huntington JL, Lawson KL, Novotny PJ, Sloan JA, Shanafelt TD. How do distress and well-being relate to medical student empathy? A multicenter study. J Gen Intern Med. 2007;22(2):177–83. 10.1007/s11606-006-0039-6. 10.1007/s11606-006-0039-6 PMC1824738 17356983

[6] Dyrbye LN, Massie FS Jr, Eacker A, Harper W, Power D, Durning SJ, Thomas MR, Moutier C, Satele D, Sloan J, Shanafelt TD. Relationship between burnout and professional conduct and attitudes among US medical students. JAMA. 2010;304(11):1173–80. 10.1001/jama.2010.1318. 10.1001/jama.2010.1318 20841530

[7] Dyrbye LN, Thomas MR, Power DV, Durning S, Moutier C, Massie FS Jr, Harper W, Eacker A, Szydlo DW, Sloan JA, Shanafelt TD. Burnout and serious thoughts of dropping out of medical school: a multi-institutional study. Acad Med. 2010;85(1):94–102. 10.1097/ACM.0b013e3181c46aad. 10.1097/ACM.0b013e3181c46aad 20042833

[8] Dyrbye LN, Sciolla AF, Dekhtyar M, Rajasekaran S, Allgood JA, Rea M, Knight AP, Haywood A, Smith S, Stephens MB. Medical School Strategies to Address Student Well-Being: A National Survey. Acad Med. 2019;94(6):861–868. 10.1097/ACM.0000000000002611. 10.1097/ACM.0000000000002611 30681453

[9] Dyrbye LN, Satele D, Shanafelt TD. Healthy Exercise Habits Are Associated With Lower Risk of Burnout and Higher Quality of Life Among U.S. Medical Students. Acad Med. 2017;92(7):1006–1011. 10.1097/ACM.0000000000001540. 10.1097/ACM.0000000000001540 28030419

[10] Howe A, Smajdor A, Stöckl A. Towards an understanding of resilience and its relevance to medical training. Med Educ. 2012;46(4):349–56. 10.1111/j.1365-2923.2011.04188.x. 10.1111/j.1365-2923.2011.04188.x 22429170

[11] Haglund ME, aan het Rot M, Cooper NS, Nestadt PS, Muller D, Southwick SM, Charney DS. Resilience in the third year of medical school: a prospective study of the associations between stressful events occurring during clinical rotations and student well-being. Acad Med. 2009;84(2):258–68. 10.1097/ACM.0b013e31819381b1. 10.1097/ACM.0b013e31819381b1 19174682

[12] Kligler B, Linde B, Katz NT. Becoming a doctor: a qualitative evaluation of challenges and opportunities in medical student wellness during the third year. Acad Med. 2013;88(4):535–40. 10.1097/ACM.0b013e3182860e6d. 10.1097/ACM.0b013e3182860e6d 23425993

[13] Swarbrick M, Gill KJ, Pratt CW (2016). Impact of peer delivered wellness coaching. Psychiatr Rehabil J.

[14] Creating a Healthier Life: A Step-By-Step Guide to Wellness. Substance Abuse Mental Health Services Administration. https://store.samhsa.gov/sites/default/files/d7/priv/sma16-4958.pdf. Accessed 29 Mar 2021.

[15] O’Brien JM, Goncin U, Ngo R, Hedlin P, Chakravarti A. Professional fulfillment, burnout, and wellness of anesthesiologists during the COVID-19 pandemic. Can J Anaesth. Published online January 20, 2021. 10.1007/s12630-021-01916-4. 10.1007/s12630-021-01916-4 PMC7817960 33475955

[16] Meo SA, Abukhalaf AA, Alomar AA, Sattar K, Klonoff DC (2020). COVID-19 Pandemic: Impact of Quarantine on Medical Students’ Mental Wellbeing and Learning Behaviors. Pak J Med Sci.

[17] Saraswathi I, Saikarthik J, Senthil Kumar K, Madhan Srinivasan K, Ardhanaari M, Gunapriya R (2020). Impact of COVID-19 outbreak on the mental health status of undergraduate medical students in a COVID-19 treating medical college: a prospective longitudinal study. PeerJ.

[18] COVID-19 Cases, Deaths, and Trends in the US | CDC COVID Data Tracker. Centers for Disease Control and Prevention. 28 March 2020. https://covid.cdc.gov/covid-data-tracker. Accessed 20 Jan 2021.

[19] Perry SP, Hardeman R, Burke SE, Cunningham B, Burgess DJ, van Ryn M (2016). The Impact of Everyday Discrimination and Racial Identity Centrality on African American Medical Student Well-Being: a Report from the Medical Student CHANGE Study. J Racial Ethn Health Disparities.

[20] Dyrbye LN, Thomas MR, Eacker A, Harper W, Massie FS, Power DV, Huschka M, Novotny PJ, Sloan JA, Shanafelt TD (2007). Race, ethnicity, and medical student well-being in the United States. Arch Intern Med.

[21] Whelan A, Prescott J, Young G, Cantanese VM, McKinney R. Guidance on Medical Students’ Participation in Direct Patient Contact Activities. Association of American Medical Colleges. 2020. https://lcme.org/wp-content/uploads/filebase/March-17-2020-Guidance-on-Mediical-Students-Clinical-Participation.pdf. Accessed 18 May 2020.

[22] O’Doherty D, Dromey M, Lougheed J, Hannigan A, Last J, McGrath D (2018). Barriers and solutions to online learning in medical education - an integrative review. BMC Med Educ.

[23] Muilenburg LY, Berge ZL (2005). Student barriers to online learning: A factor analytic study. Distance Educ.

[24] Tuition and Student Fees Reports. In: Medical Education. Association of American Medical Colleges. 2020. https://www.aamc.org/data-reports/reporting-tools/report/tuition-and-student-fees-reports. Accessed 11 June 2020.

[25] Physician Education Debt and the Cost to Attend Medical School | 2020 Update. Association of American Medical Colleges. 2020. https://store.aamc.org/downloadable/download/sample/sample_id/368/. Accessed 5 Apr 2020.

[26] Pant J, Pant MK, Naithani M. Contribution and dilemmas of Medical undergraduate students in combating disease outbreaks: COVID 19 and previous outbreaks. Adv Med Educ Pract. 2020;11:661–667. 10.2147/AMEP.S265558. 10.2147/AMEP.S265558 PMC7535110 33061736

[27] Rose S (2020). Medical Student Education in the Time of COVID-19. JAMA.

[28] Krieger P, Goodnough A. Medical Students, Sidelined for Now, Find New Ways to Fight Coronavirus. The New York Times. 2020. https://www.nytimes.com/2020/03/23/health/medical-students-coronavirus.html. Accessed 10 June 2020.

[29] Miller DG, Pierson L, Doernberg S (2020). The Role of Medical Students During the COVID-19 Pandemic. Ann Intern Med.

[30] Thoits PA (2012). Role-Identity Salience, Purpose and Meaning in Life, and Well-Being among Volunteers. Soc Psychol Q.

[31] Vignoles VL, Regalia C, Manzi C, Golledge J, Scabini E (2006). Beyond self-esteem: influence of multiple motives on identity construction. J Pers Soc Psychol.

[32] Slivkoff MD, Johnson C, Tackett S. First-Year Medical Student Experiences Adjusting to the Immediate Aftermath of COVID-19. MedSciEduc. Published online January 22, 2021. 10.1007/s40670-021-01213-1. 10.1007/s40670-021-01213-1 PMC7822582 33520395

[33] Murphy B. Delays, miscommunications add even more stress to USMLE Step exams. American Medical Association. 2020. https://www.ama-assn.org/residents-students/usmle/delays-miscommunications-add-even-more-stress-usmle-step-exams. Accessed 10 June 2020.

[34] Murphy B. COVID-19 and USMLE testing: 4 key questions for students, residents. American Medical Association. 2020. https://www.ama-assn.org/residents-students/usmle/covid-19-and-usmle-testing-4-key-questions-students-residents. Accessed 10 June 2020.

[35] Strowd RE, Lambros A. Impacting student anxiety for the USMLE Step 1 through process-oriented preparation. Med Educ Online. 2010;15. 10.3402/meo.v15i0.4880. 10.3402/meo.v15i0.4880 PMC2830045 20198129

[36] Final Report and Recommendations for Medical Education Institutions of LCME-Accredited, U.S. Osteopathic, and Non-U.S. Medical School Applicants. Association of American Medical Colleges. 2020. https://www.aamc.org/system/files/2020-05/covid19_Final_Recommendations_Executive%20Summary_Final_05112020.pdf. Accessed 17 May 2020.

[37] Slavin SJ, Schindler DL, Chibnall JT (2014). Medical Student Mental Health 3.0: Improving Student Wellness Through Curricular Changes. Acad Med.

[38] Chen P, Mao L, Nassis GP, Harmer P, Ainsworth BE, Li F (2020). Coronavirus disease (COVID-19): The need to maintain regular physical activity while taking precautions. J Sport Health Sci.

[39] Hu FB, Li TY, Colditz GA, Willett WC, Manson JE (2003). Television watching and other sedentary behaviors in relation to risk of obesity and type 2 diabetes mellitus in women. JAMA.

[40] Liu M, Wu L, Yao S (2016). Dose–response association of screen time-based sedentary behaviour in children and adolescents and depression: a meta-analysis of observational studies. Br J Sports Med.

[41] Teychenne M, Costigan SA, Parker K (2015). The association between sedentary behaviour and risk of anxiety: a systematic review. BMC Public Health.

[42] Keren D, Lockyer J, Ellaway RH (2017). Social studying and learning among medical students: a scoping review. Perspect Med Educ.

[43] Champaloux EP, Keeley MG. The impact of learning communities on interpersonal relationships among medical students. Med Educ Online. 2016;21. 10.3402/meo.v21.32958. 10.3402/meo.v21.32958 PMC5093321 27806828

