# Supplementary material for: Medical student wellness in the United States during the COVID-19 pandemic: a nationwide survey
Source: BMC Med Educ. 2021 Jul 26;21:401. doi: 10.1186/s12909-021-02837-y (PMC8312706; doi:10.1186/s12909-021-02837-y)
Supplement: Supplementary file 1 — Additional file 1: [file 12909_2021_2837_MOESM1_ESM.pdf]

Medical Student Wellness During the COVID-19 Pandemic

Dear Participant,

Andrea Wakim, Louis Nikolis, and Dr. Prempreet Bajaj invite you to participate in the attached survey. Your participation is completely voluntary. There is no compensation and there is no risk of participation. The survey should require 5-10 minutes of your time. All answers are confidential. All answers are anonymous. Please do not include your name.

If you choose to participate, please answer all questions as honestly as possible and screenshot or print out the confirmation of completion upon finishing the survey. Completion of the survey will indicate your willingness to participate.

We thank you for your participation.

If you would like additional information or have questions, please contact Dr. Prempreet Bajaj, D.O., faculty advisor of this project, at pbajaj@lumc.edu.

Sincerely,

Dr. Prempreet Bajaj, DO
Associate Professor, Physical Medicine and Rehabilitation
Department of Orthopaedics and Rehabilitation
Loyola University Stritch School of Medicine

Medical Student Wellness During the COVID-19 Pandemic

Demographics

* 1. Please indicate your year in medical school.

- ○ MS1
- ○ MS2
- ○ MS3
- ○ MS4

* 2. Please indicate your sex.

- ○ Male
- ○ Female
- ○ Non-binary
- ○ Other
- ○ I prefer not to say

* 3. Please indicate your ethnicity.

- ○ White/Caucasian
- ○ Hispanic/Latino
- ○ Black/African American
- ○ Asian
- ○ Native American
- ○ Other
- ○ Prefer not to say

* 4. What school do you attend?

____

* 5. Please write the city, state your school is located in.

____

* 6. Please indicate yes or no for the following questions:

	Yes	No
Has your school suspended in-person meetings?	◯	◯
Are you taking a required class or clerkship?	◯	◯
Are you taking an elective class or clerkship?	◯	◯
Are your classes or clerkships utilizing recorded lectures?	◯	◯
Are your classes or clerkships utilizing live online lectures?	◯	◯
Do your classes or clerkships have required exams?	◯	◯
Are you currently assisting in in-person patient care?	◯	◯
Are you currently participating in in-person care of COVID-19 patients?	◯	◯
Are you currently assisting in other COVID-19 relief efforts? (ex. working at a COVID-19 hotline, volunteering for a community organization, making cloth masks, etc.)	◯	◯

Medical Student Wellness During the COVID-19 Pandemic

Wellness Measures

Please answer the following questions based on your typical week.

* 7. How much time are you studying per day?

	Not at all	0-2 hours	2-4 hours	4-6 hours	6-8 hours	>8 hours
Before COVID-19						
During COVID-19						

* 8. How many hours do you sleep per night?

	<4 hours	4-6 hours	6-8 hours	8-10 hours	>10 hours
Before COVID-19					
During COVID-19					

* 9. How often are you exercising per week?

	Not at all	1-2 days per week	3-4 days per week	5-6 days per week	7 days per week
Before COVID-19					
During COVID-19					

* 10. How often do you speak to your friends/family per week?

	Not at all	1-2 days per week	3-4 days per week	5-6 days per week	7 days per week
Before COVID-19					
During COVID-19					

* 11. How often do you worry about finances?

	Not at all	1-2 days per week	3-4 days per week	5-6 days per week	7 days per week
Before COVID-19					
During COVID-19					

* 12. How often do you reflect on your sense of purpose?

	Not at all	1-2 days per week	3-4 days per week	5-6 days per week	7 days per week
Before COVID-19					
During COVID-19					

* 13. Where do you spend the majority of your time during the week?

	At home	Outdoors	At school/work	At a friend/significant other's home	Other
Before COVID-19	◯	◯	◯	◯	◯
During COVID-19	◯	◯	◯	◯	◯

* 14. How do you interact with friends/family? (Select all that apply.)

	In person	Text	Facetime	Phone-call	Email	N/A
Before COVID-19	☐	☐	☐	☐	☐	☐
During COVID-19	☐	☐	☐	☐	☐	☐

* 15. What strengthens your sense of purpose? (Select all that apply.)

	School/Work	Friends/Family	Hobbies	Community Service	Religion	Reflection	Other
Before COVID-19	☐	☐	☐	☐	☐	☐	☐
During COVID-19	☐	☐	☐	☐	☐	☐	☐

Medical Student Wellness During the COVID-19 Pandemic

Wellness Measures Part 2 of 2

Please answer the following questions based on your typical week.

* 16. I am satisfied with my sleep.

	Strongly Disagree	Disagree	Neutral	Agree	Strongly Agree
Before COVID-19	○	○	○	○	○
During COVID-19	○	○	○	○	○

* 17. I am satisfied with my level of exercise.

	Strongly Disagree	Disagree	Neutral	Agree	Strongly Agree
Before COVID-19	○	○	○	○	○
During COVID-19	○	○	○	○	○

* 18. I am satisfied with my nutritional intake.

	Strongly Disagree	Disagree	Neutral	Agree	Strongly Agree
Before COVID-19	○	○	○	○	○
During COVID-19	○	○	○	○	○

* 19. I am satisfied with my work/school situation.

	Strongly Disagree	Disagree	Neutral	Agree	Strongly Agree
Before COVID-19	○	○	○	○	○
During COVID-19	○	○	○	○	○

20. I feel confident in my medical education.

	Strongly Disagree	Disagree	Neutral	Agree	Strongly Agree
Before COVID-19	○	○	○	○	○
During COVID-19	○	○	○	○	○

21. I feel comfortable in my ability to provide patient care.

	Strongly Disagree	Disagree	Neutral	Agree	Strongly Agree
Before COVID-19	○	○	○	○	○
During COVID-19	○	○	○	○	○

* 22. I feel stressed.

	Strongly Disagree	Disagree	Neutral	Agree	Strongly Agree
Before COVID-19	○	○	○	○	○
During COVID-19	○	○	○	○	○

* 23. I feel anxious.

	Strongly Disagree	Disagree	Neutral	Agree	Strongly Agree
Before COVID-19	○	○	○	○	○
During COVID-19	○	○	○	○	○

* 24. I feel depressed.

	Strongly Disagree	Disagree	Neutral	Agree	Strongly Agree
Before COVID-19	○	○	○	○	○
During COVID-19	○	○	○	○	○

* 25. I feel burned out.

	Strongly Disagree	Disagree	Neutral	Agree	Strongly Agree
Before COVID-19	○	○	○	○	○
During COVID-19	○	○	○	○	○

* 26. I have enough time to study.

	Strongly Disagree	Disagree	Neutral	Agree	Strongly Agree
Before COVID-19	○	○	○	○	○
During COVID-19	○	○	○	○	○

* 27. I am worried about my grades.

	Strongly Disagree	Disagree	Neutral	Agree	Strongly Agree
Before COVID-19	○	○	○	○	○
During COVID-19	○	○	○	○	○

* 28. I feel supported by my social environment.

	Strongly Disagree	Disagree	Neutral	Agree	Strongly Agree
Before COVID-19	○	○	○	○	○
During COVID-19	○	○	○	○	○

* 29. I enjoy my work/school-work.

	Strongly Disagree	Disagree	Neutral	Agree	Strongly Agree
Before COVID-19	◯	◯	◯	◯	◯
During COVID-19	◯	◯	◯	◯	◯

* 30. I feel satisfied with my financial situation.

	Strongly Disagree	Disagree	Neutral	Agree	Strongly Agree
Before COVID-19	◯	◯	◯	◯	◯
During COVID-19	◯	◯	◯	◯	◯

* 31. I feel comfortable in my daily environment

	Strongly Disagree	Disagree	Neutral	Agree	Strongly Agree
Before COVID-19	◯	◯	◯	◯	◯
During COVID-19	◯	◯	◯	◯	◯

* 32. I am satisfied with my sense of purpose.

	Strongly Disagree	Disagree	Neutral	Agree	Strongly Agree
Before COVID-19	◯	◯	◯	◯	◯
During COVID-19	◯	◯	◯	◯	◯

* 33. How would you rate your energy levels?

	Very Low	Low	Neutral	High	Very High
Before COVID-19	◯	◯	◯	◯	◯
During COVID-19	◯	◯	◯	◯	◯

* 34. How would you rate your OVERALL level of wellness? (0 = least well; 10 = most well)

	0	1	2	3	4	5	6	7	8	9	10
Before COVID-19	◯	◯	◯	◯	◯	◯	◯	◯	◯	◯	◯
During COVID-19	◯	◯	◯	◯	◯	◯	◯	◯	◯	◯	◯

35. Please share any other comments regarding wellness which were not addressed in this survey. (Optional)
