# Supplementary material for: Medical student wellness in the United States during the COVID-19 pandemic: a nationwide survey
Source: BMC Med Educ. 2021 Jul 26;21:401. doi: 10.1186/s12909-021-02837-y (PMC8312706; doi:10.1186/s12909-021-02837-y)
Supplement: Supplementary file 2 — Additional file 2: [file 12909_2021_2837_MOESM2_ESM.docx]

**Supplementary File 2.** Summary frequencies for Table 3

|  | | **Survey Period** | | | |
| --- | --- | --- | --- | --- | --- |
|  |  | **Pre-COVID** | | **During COVID** | |
|  |  | **Count** | **%** | **Count** | **%** |
| **How many hours do you sleep per night?** | **<4 hours** | 8 | 0.6% | 13 | 1.0% |
|  | **4-6 hours** | 198 | 15.0% | 124 | 9.4% |
|  | **6-8 hours** | 960 | 72.8% | 668 | 50.8% |
|  | **8-10 hours** | 152 | 11.5% | 475 | 36.1% |
|  | **>10 hours** | 1 | 0.1% | 35 | 2.7% |
|  | **Total** | 1319 | 100.0% | 1315 | 100.0% |
| **I am satisfied with my sleep** | **Strongly Disagree** | 68 | 5.3% | 115 | 9.0% |
|  | **Disagree** | 306 | 23.8% | 262 | 20.4% |
|  | **Neutral** | 320 | 24.8% | 204 | 15.9% |
|  | **Agree** | 490 | 38.0% | 519 | 40.4% |
|  | **Strongly Agree** | 104 | 8.1% | 184 | 14.3% |
|  | **Total** | 1288 | 100.0% | 1284 | 100.0% |
| **How would you rate your energy levels?** | **Very Low** | 21 | 1.6% | 152 | 11.9% |
|  | **Low** | 187 | 14.5% | 439 | 34.2% |
|  | **Neutral** | 515 | 40.0% | 418 | 32.6% |
|  | **High** | 504 | 39.1% | 241 | 18.8% |
|  | **Very High** | 61 | 4.7% | 32 | 2.5% |
|  | **Total** | 1288 | 100.0% | 1282 | 100.0% |
| **I am satisfied with my nutritional intake** | **Strongly Disagree** | 52 | 4.0% | 116 | 9.0% |
|  | **Disagree** | 219 | 17.0% | 286 | 22.2% |
|  | **Neutral** | 290 | 22.5% | 246 | 19.1% |
|  | **Agree** | 623 | 48.4% | 518 | 40.3% |
|  | **Strongly Agree** | 104 | 8.1% | 120 | 9.3% |
|  | **Total** | 1288 | 100.0% | 1286 | 100.0% |
| **How often are you exercising per week?** | **Not at all** | 191 | 14.5% | 221 | 16.7% |
|  | **1-2 days per week** | 380 | 28.8% | 315 | 23.9% |
|  | **3-4 days per week** | 437 | 33.1% | 340 | 25.8% |
|  | **5-6 days per week** | 259 | 19.6% | 334 | 25.3% |
|  | **7 days per week** | 53 | 4.0% | 110 | 8.3% |
|  | **Total** | 1320 | 100.0% | 1320 | 100.0% |
| **I am satisfied with my level of exercise** | **Strongly Disagree** | 152 | 11.8% | 284 | 22.1% |
|  | **Disagree** | 395 | 30.7% | 322 | 25.0% |
|  | **Neutral** | 234 | 18.2% | 154 | 12.0% |
|  | **Agree** | 374 | 29.0% | 364 | 28.3% |
|  | **Strongly Agree** | 133 | 10.3% | 162 | 12.6% |
|  | **Total** | 1288 | 100.0% | 1286 | 100.0% |
| **How often do you speak to your friends/family per week?** | **Not at all** | 17 | 1.3% | 11 | 0.8% |
|  | **1-2 days per week** | 424 | 32.1% | 183 | 13.9% |
|  | **3-4 days per week** | 313 | 23.7% | 327 | 24.8% |
|  | **5-6 days per week** | 215 | 16.3% | 292 | 22.1% |
|  | **7 days per week** | 352 | 26.6% | 506 | 38.4% |
|  | **Total** | 1321 | 100.0% | 1319 | 100.0% |
| **I feel supported by my social environment** | **Strongly Disagree** | 16 | 1.2% | 64 | 5.0% |
|  | **Disagree** | 62 | 4.8% | 193 | 15.0% |
|  | **Neutral** | 175 | 13.6% | 253 | 19.7% |
|  | **Agree** | 782 | 60.7% | 567 | 44.2% |
|  | **Strongly Agree** | 253 | 19.6% | 207 | 16.1% |
|  | **Total** | 1288 | 100.0% | 1284 | 100.0% |
| **How often do you reflect on your sense of purpose?** | **Not at all** | 253 | 19.2% | 152 | 11.5% |
|  | **1-2 days per week** | 625 | 47.3% | 293 | 22.2% |
|  | **3-4 days per week** | 272 | 20.6% | 387 | 29.3% |
|  | **5-6 days per week** | 79 | 6.0% | 254 | 19.3% |
|  | **7 days per week** | 92 | 7.0% | 233 | 17.7% |
|  | **Total** | 1321 | 100.0% | 1319 | 100.0% |
| **I am satisfied with my sense of purpose** | **Strongly Disagree** | 8 | 0.6% | 48 | 3.7% |
|  | **Disagree** | 57 | 4.4% | 259 | 20.1% |
|  | **Neutral** | 248 | 19.3% | 363 | 28.2% |
|  | **Agree** | 774 | 60.1% | 489 | 38.0% |
|  | **Strongly Agree** | 201 | 15.6% | 127 | 9.9% |
|  | **Total** | 1288 | 100.0% | 1286 | 100.0% |
| **I feel comfortable in my daily environment** | **Strongly Disagree** | 9 | 0.7% | 46 | 3.6% |
|  | **Disagree** | 40 | 3.1% | 194 | 15.1% |
|  | **Neutral** | 172 | 13.4% | 251 | 19.5% |
|  | **Agree** | 864 | 67.1% | 611 | 47.5% |
|  | **Strongly Agree** | 203 | 15.8% | 183 | 14.2% |
|  | **Total** | 1288 | 100.0% | 1285 | 100.0% |
| **How often do you worry about finances?** | **Not at all** | 497 | 37.6% | 402 | 30.5% |
|  | **1-2 days per week** | 537 | 40.7% | 432 | 32.7% |
|  | **3-4 days per week** | 172 | 13.0% | 223 | 16.9% |
|  | **5-6 days per week** | 47 | 3.6% | 118 | 8.9% |
|  | **7 days per week** | 68 | 5.1% | 145 | 11.0% |
|  | **Total** | 1321 | 100.0% | 1320 | 100.0% |
| **I feel satisfied with my financial situation** | **Strongly Disagree** | 75 | 5.8% | 155 | 12.1% |
|  | **Disagree** | 233 | 18.1% | 264 | 20.5% |
|  | **Neutral** | 416 | 32.3% | 371 | 28.8% |
|  | **Agree** | 447 | 34.7% | 372 | 28.9% |
|  | **Strongly Agree** | 117 | 9.1% | 124 | 9.6% |
|  | **Total** | 1288 | 100.0% | 1286 | 100.0% |
| **How much time are you studying per day?** | **Not at all** | 33 | 2.5% | 73 | 5.6% |
|  | **0-2 hours** | 138 | 10.4% | 138 | 10.5% |
|  | **2-4 hours** | 286 | 21.7% | 195 | 14.8% |
|  | **4-6 hours** | 258 | 19.5% | 305 | 23.2% |
|  | **6-8 hours** | 283 | 21.4% | 293 | 22.3% |
|  | **>8 hours** | 323 | 24.5% | 311 | 23.7% |
|  | **Total** | 1321 | 100.0% | 1315 | 100.0% |
| **I have enough time to study** | **Strongly Disagree** | 67 | 5.2% | 40 | 3.1% |
|  | **Disagree** | 345 | 26.8% | 128 | 10.0% |
|  | **Neutral** | 329 | 25.5% | 172 | 13.4% |
|  | **Agree** | 480 | 37.3% | 616 | 47.9% |
|  | **Strongly Agree** | 67 | 5.2% | 330 | 25.7% |
|  | **Total** | 1288 | 100.0% | 1286 | 100.0% |
| **I am worried about my grades** | **Strongly Disagree** | 117 | 9.1% | 138 | 10.7% |
|  | **Disagree** | 301 | 23.4% | 297 | 23.1% |
|  | **Neutral** | 251 | 19.5% | 219 | 17.0% |
|  | **Agree** | 486 | 37.7% | 391 | 30.4% |
|  | **Strongly Agree** | 133 | 10.3% | 241 | 18.7% |
|  | **Total** | 1288 | 100.0% | 1286 | 100.0% |
| **I feel confident in my medical education** | **Strongly Disagree** | 14 | 1.1% | 139 | 10.8% |
|  | **Disagree** | 79 | 6.1% | 358 | 27.8% |
|  | **Neutral** | 216 | 16.8% | 379 | 29.5% |
|  | **Agree** | 776 | 60.2% | 350 | 27.2% |
|  | **Strongly Agree** | 203 | 15.8% | 60 | 4.7% |
|  | **Total** | 1288 | 100.0% | 1286 | 100.0% |
| **I feel comfortable in my ability to provide patient care** | **Strongly Disagree** | 14 | 1.1% | 89 | 6.9% |
|  | **Disagree** | 130 | 10.1% | 333 | 26.0% |
|  | **Neutral** | 354 | 27.5% | 459 | 35.8% |
|  | **Agree** | 663 | 51.6% | 330 | 25.7% |
|  | **Strongly Agree** | 125 | 9.7% | 72 | 5.6% |
|  | **Total** | 1286 | 100.0% | 1283 | 100.0% |
| **I am satisfied with my work/school situation** | **Strongly Disagree** | 33 | 2.6% | 300 | 23.4% |
|  | **Disagree** | 107 | 8.3% | 464 | 36.1% |
|  | **Neutral** | 261 | 20.3% | 248 | 19.3% |
|  | **Agree** | 706 | 54.8% | 229 | 17.8% |
|  | **Strongly Agree** | 181 | 14.1% | 43 | 3.3% |
|  | **Total** | 1288 | 100.0% | 1284 | 100.0% |
| **I enjoy my work/school-work** | **Strongly Disagree** | 20 | 1.6% | 94 | 7.3% |
|  | **Disagree** | 55 | 4.3% | 280 | 21.8% |
|  | **Neutral** | 220 | 17.1% | 357 | 27.8% |
|  | **Agree** | 768 | 59.6% | 444 | 34.5% |
|  | **Strongly Agree** | 225 | 17.5% | 111 | 8.6% |
|  | **Total** | 1288 | 100.0% | 1286 | 100.0% |
| **I feel stressed** | **Strongly Disagree** | 14 | 1.1% | 22 | 1.7% |
|  | **Disagree** | 88 | 6.8% | 76 | 5.9% |
|  | **Neutral** | 198 | 15.4% | 123 | 9.6% |
|  | **Agree** | 808 | 62.8% | 475 | 37.0% |
|  | **Strongly Agree** | 179 | 13.9% | 589 | 45.8% |
|  | **Total** | 1287 | 100.0% | 1285 | 100.0% |
| **I feel anxious** | **Strongly Disagree** | 32 | 2.5% | 31 | 2.4% |
|  | **Disagree** | 191 | 14.8% | 125 | 9.7% |
|  | **Neutral** | 340 | 26.4% | 146 | 11.4% |
|  | **Agree** | 596 | 46.3% | 450 | 35.0% |
|  | **Strongly Agree** | 129 | 10.0% | 534 | 41.5% |
|  | **Total** | 1288 | 100.0% | 1286 | 100.0% |
| **I feel depressed** | **Strongly Disagree** | 194 | 15.1% | 158 | 12.3% |
|  | **Disagree** | 488 | 37.9% | 353 | 27.4% |
|  | **Neutral** | 362 | 28.1% | 253 | 19.7% |
|  | **Agree** | 212 | 16.5% | 346 | 26.9% |
|  | **Strongly Agree** | 32 | 2.5% | 176 | 13.7% |
|  | **Total** | 1288 | 100.0% | 1286 | 100.0% |
| **I feel burned out** | **Strongly Disagree** | 80 | 6.2% | 99 | 7.7% |
|  | **Disagree** | 366 | 28.4% | 295 | 23.0% |
|  | **Neutral** | 343 | 26.6% | 241 | 18.8% |
|  | **Agree** | 395 | 30.7% | 341 | 26.5% |
|  | **Strongly Agree** | 104 | 8.1% | 309 | 24.0% |
|  | **Total** | 1288 | 100.0% | 1285 | 100.0% |
